# RETRACTED ARTICLE: Comparative genomics of two *Shewanella xiamenensis* strains isolated from a pilgrim before and during travels to the Hajj

**DOI:** 10.1186/s13099-021-00404-w

**Published:** 2021-02-09

**Authors:** Thongpan Leangapichart, Linda Hadjadj, Philippe Gautret, Jean-Marc Rolain

**Affiliations:** 1 Aix Marseille Univ, IRD, APHM, MEPHI, IHU Méditerranée Infection, Faculté de Médecine Et de Pharmacie, 19-21 Boulevard Jean Moulin, 13385 Marseille CEDEX 05, France; 2 IHU Méditerranée Infection, 19-21 Boulevard Jean Moulin, 13385 Marseille CEDEX 05, France

The Editors have retracted this article because the authors confirmed that they did not obtain informed consent to publish the description of the case. The content of this article is no longer available online to protect patient confidentiality.

The authors did not respond to correspondence from the Publisher regarding this retraction.

